# Cremation

**Published:** 1899-04-15

**Authors:** 


### The Hospital

A Journal of

The Medical Sciences and Hospital Administration.

Vol. XXVI.—No. 655.] Edited by Sir Henry Burdett, K.C.B., and Solomon C. Smith, M.D., M.R.C.P. [April 15, 1899.

### Cremation.

On the occasion of the annual meeting of the Cremation Society of England, which was held last month, Sir Henry Thompson stated that the society had now been in existence for twenty-five years, and he took the opportunity thus presented of giving an interesting review of the progress which cremation had made in England during that period, pointing out at the same time the directions in which advance should be aimed at in the near future, one of which was the erection of a crematorium much nearer to London than the one which is at present in use. We are by no means thoroughgoing cremationists, but, while we think it probable that methods of burial might be much improved—so much, in fact, as to do away with much of the evil which now attaches to them—we cannot but admit that cremation also is as yet far from perfection. Of course, the whole question is still terribly mixed up with sentiment, but if we put sentiment on one side for the moment, and merely consider what is the best method of disposing of the bodies of the dead, we find it difficult to separate that question from the larger one, What is the best method of disposing of those other forms of decomposable organic matter which tend to accumulate in every town, and must for sanitary reasons be got rid of? Destruction of organic waste material by means of heat—in other words, cremation—is the method which seems according to our present knowledge the least injurious to the living, but it must always be a wasteful and expensive process unless proper means be taken to utilise the great heat which has of necessity to be employed if cremation is to be conducted in a thoroughly effectual manner. For the proper cremation of animal substances without the production of a nuisance something much more than mere burning is required. Such a temperature is necessary as will not merely burn up the solid body into gases, but will also consume these vilely odorous gases into harmless compounds. To heat a furnace so as to produce such temperatures for each single cremation, and then to throw away that heat unused is of course a wasteful process. But where the destruction of animal substances has to be carried out continuously, as in modern refuse destructors, there is no difficulty; not only is the heat which is used made to give up its energy in the form of power, but every particle of refuse consumed adds its quota to the sum of the heat produced.

Gentle reader, do not be shocked! We are not suggesting, as some flippant persons have done, that our dead should be sent to the gas works and thus turned to useful purposes. Far from us be such a thought. We still think that in country places where there is room, and where burial can be decently performed, to place the dead under the sod and to leave Nature with her worms and microbes to complete the process of disintegration is the simplest plan, although full of nastiness. But in towns and in all places where human beings are so crowded together that this cannot be done with safety to the living, and where cremation is offered as a substitute, the plain question—from the point of view of science—is, How can this process be carried out most effectually, with the greatest economy, and with the least nuisance? and we can have no doubt about the answer, namely, that this process of driving off from the bodies of the dead those carbonaceous and nitrogenous elements on which their offensiveness depends can best be done in connection with, although as a separate department of, those great destructors which every community will soon find itself compelled to possess. Nor need this be looked upon as desecration of the dead. Between death and burial many things are done which no one cares to think of or to inquire about too closely. We give our dead into the hands of the undertakers and their satellites, and in due season they give us back a box with a name attached, which we bury with all reverence. But the intervening washing and shaving and dressing, the screwing down and the soldering up, the transit by rail or, perhaps, by steamer from distant country, the handling by unsympathetic people, and in some cases the horrid process of embalming the body, are all things the very thought of which we shrink from. In fact, we leave them to the undertaker. And would it not be a more reverent treatment of the dead if the same hands that had gently nursed her on her bed gently lifted her just as she lay, and placed her in a simple wicker or light wooden coffin, and then handed over this casket to the undertaker? Rough men should never touch, should never even look upon the dead face of the loved one; but the coffin should be taken to the crematorium, whence would be returned what, although but a heap of ashes, would be the mortal remains of the deceased as truly as if it were the corpse itself, and these remains the mourners could themselves commit to the grave with all reverence and propriety. Where does the desecration come in? We would have the same tenderness used to the dead as to the living, and we would let the undertaker handle the coffin rather than the corpse. Surely this is the gentlest plan, and far better than that cremation should, as at present, be elevated into a rite, and that the mourners should sit in however beautiful a temple, conscious that the furnace is at work, and that the stokers are busy behind the doors.

